# 5-Lipoxygenase DNA Methylation and mRNA Content in the Brain and Heart of Young and Old Mice

**DOI:** 10.1155/2009/209596

**Published:** 2009-12-13

**Authors:** Svetlana Dzitoyeva, Marta Imbesi, Louisa W. Ng, Hari Manev

**Affiliations:** The Psychiatric Institute, Department of Psychiatry, University of Illinois at Chicago, 1601 West Taylor Street, MC912, Chicago, IL 60612, USA

## Abstract

The expression of 5-lipoxygenase (5-LOX) is affected by aging and regulated by epigenetic mechanisms including DNA methylation. We used methylation-sensitive restriction endonucleases (AciI, BstUI, HpaII, and HinP1I) to assess 5-LOX DNA methylation in brain and heart tissue samples from young (2 months) and old (22 months) mice. We also measured mRNA content for 5-LOX and the DNA methyltransferases DNMT1 and DNMT3a. In young mice, the 5-LOX mRNA content was significantly greater in the heart compared to the brain; 5-LOX DNA methylation was lower, except in the AciI assay in which it was higher in the heart. Aging decreased 5-LOX mRNA content in the heart and increased it in the brain. Aging also increased 5-LOX DNA methylation and this effect was site- (i.e., enzyme) and tissue-specific. Generally, DNMT1 and DNMT3a mRNA content was lower in the brain regions compared to the heart; the only effect of aging was observed in the mRNA content of DNMT3a, which was decreased in the heart of old mice. These results indicate a complex tissue-specific and aging-dependent interplay between the DNA methylation system and 5-LOX mRNA content. Interpretation of this data must take into account that the tissue samples contained a mixture of various cell types.

## 1. Introduction

5-lipoxygenase (5-LOX; EC 1.13.11.34; encoded by the ALOX5 gene) is the key enzyme in the biosynthesis of lipid signaling molecules, that is, the inflammatory leukotrienes and the anti-inflammatory lipoxins [1, 2]. The 5-LOX system is considered one of the key players in inflammation [3], an important pathway in the cardiovascular system (particularly in atherosclerosis) [4, 5], and a putative modulator of central nervous system (CNS) functioning and pathobiology [6–8]. Moreover, the 5-LOX pathway has been considered in mechanisms of neural plasticity ranging from neurogenesis and neural differentiation [9] to regulation of synaptic plasticity [10, 11]. For example, metabolites derived from 5-LOX activity have been implicated in the expression of long-term depression in hippocampal slices [10]. 

 It is believed that a nonleukocyte synthesis of leukotrienes does not require 5-LOX and that in the CNS and in the heart, leukotrienes can be produced via a transcellular biosynthetic pathway [12, 13]. Nevertheless, there is evidence for 5-LOX expression in these organs/systems [6, 14–16]. Thus, regulation of the brain expression of 5-LOX participates in mechanisms of neuroprotection [8]. In the CNS of rats and mice, 5-LOX expression increases during aging [14, 17] and in brain ischemia [18, 19]. In the human brain, 5-LOX protein content is elevated in Alzheimer's disease [7, 20] and in response to ischemia [21]. In the heart, 5-LOX content appears to remain stable during cardiac remodeling [16] but can be altered by dietary modifications [22]. Furthermore, in the heart, that is, myocardial cells, drugs such as statins and thiazolidinediones trigger anti-inflammatory processes and increase the production of anti-inflammatory lipoxins depending on 5-LOX, particularly the status of 5-LOX phosphorylation [15, 23]. 

 5-LOX gene expression is regulated by epigenetic mechanisms including modifications of DNA methylation [1, 24–26]. Epigenetics encompasses the modification of chromatin structure that leads to regulation of the gene expression and phenotype. Epigenetic modifications are further influenced by the environment (“life experience”) and are typically long lasting and sometimes heritable. Recently, epigenetic mechanisms have been implicated in the pathobiology of psychiatric disorders [27]. Thus, DNA methylation at the sites of CpG dinucleotides has been implicated in gene regulation in virtually all tissues, including the brain [28]. Siegmund et al. [28] studied human cerebral cortex samples in aging subjects and found a progressive aging-associated rise in the DNA methylation of 5′ CpG islands of certain CNS genes, typically in conjunction with declining levels of their corresponding mRNAs. These authors concluded that DNA methylation is dynamically regulated in the human cerebral cortex throughout the lifespan, involves differentiated neurons, and affects a substantial portion of genes predominantly by an age-related increase. In addition, data are available on epigenetic mechanisms and cardiovascular gene regulation. For example, it was observed that the genomic DNA methylation in patients with coronary artery disease is significantly higher than in controls [29]. 

 It has been hypothesized that the 5-LOX pathway may be involved in the comorbidity of certain heart and brain disorders [30, 31]. Furthermore, it was proposed that comparative studies of heart and brain tissues from the same subjects would improve efforts in this area of research [32]. Here, we investigated whether aging affects brain and heart 5-LOX mRNA levels and DNA methylation. Because mice are used in many 5-LOX-related experimental models, including 5-LOX-deficient mice, we investigated mouse tissue samples. However, a conundrum of epigenetic research of CNS- and heart-expressed genes lies not only in the cell-specificity of epigenetic mechanisms, but also in their species-specificity. Thus, the human 5-LOX gene is on chromosome 10, whereas in the mouse it is on chromosome 6. Comparing the DNA regions of the 5-LOX promoter and exon 1 (1844 nt, human; 1937 nt, mouse), we found 132 CpG dinucleotides in the human and only 59 in the mouse gene promoter regions. To study the methylation status of this region, we employed our recently developed methylation-sensitive restriction endonuclease assay [33]. Since DNA methylation depends on DNA-methyltransferases (DNMTs), we investigate the tissue content of mRNAs for DNMT1 and DNMT3a, the *maintenance,* and the *de novo* DNMTs, respectively [34]. 

## 2. Material and Methods

### 2.1. Tissue Samples

Two-month-old (young) and 22-month-old (old) C57BL/6J male mice were obtained from the National Institute on Aging (Bethesda, MD). They were housed in groups of 4–6 in a temperature controlled room and had free access to laboratory chow and water. Cerebellum, frontal cortex, hippocampus, and heart (the apex portion was selected as a source of uniform ventricular myocytes [35]) tissues were obtained following lethal anesthesia (160 mg/kg ketamine; Sigma, St. Louis, MO) and transcardial perfusion with 0.9% ice-cold saline to remove the circulating blood cells (i.e., until the outflow from the right atrium was clear). The experimental protocol was approved by the Institutional Animal Care Committee.

### 2.2. RNA Isolation and Real-Time PCR (qPCR)

The total RNA was extracted using the chemical isolation method with TRIzol reagent (Invitrogen, Carlsbad, CA) following the manufacturer's instructions. To eliminate possible DNA contamination, RNA samples were treated with a DNase reagent, DNA-free (Ambion, Inc., Austin, TX). Total RNA (3 *μ*g) was reverse transcribed with 200 U of cloned Moloney Murine Leukemia Virus (M-MLV) reverse transcriptase (Gibco BRL, Carlsbad, CA). The qPCR was performed in a Stratagene Mx3005P QPCR System (Stratagene, La Jolla, CA) using Maxima SYBR Green qPCR Master Mix (Fermentas, Glen Burnie, MD) in a two-step cycling protocol as described by the manufacturer. There was an initial 5 minutes denaturing followed by 40 cycles of denaturing at 95°C for 15 seconds and annealing/elongation at 60°C for 1 minute. Table 1 shows primer sequences used to detect 5-LOX, DNMTs, and cyclophilin mRNAs (primers were purchased from Integrated DNA Technologies, Inc., Coralville, IA). For validation purposes, the amplification products of all above-noted genes were run on agarose gels. For each product, we obtained one single band of the expected size (data not shown). Furthermore, the 5-LOX PCR product was verified by sequencing. Reactions were performed using three different concentrations (12.5, 25, 50 ng) of reverse transcribed material (in duplicate for each sample). The qPCR results were normalized against the corresponding cyclophilin contents. Data are presented in units calculated as a coefficient of variation 2^−[∆Ct*  *(target)*  *−*  *∆Ct*  *(input)]^ [36].

### 2.3. 5-LOX DNA Methylation Assay

**Figure 1:**
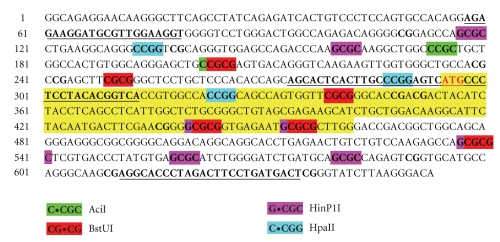
The sequence of the mouse 5-LOX promoter-5′ UTR and the first exon-intron region subjected to the methylation-sensitive endonuclease assay with the four endonucleases—AciI, BstUI, HinP1I, and HpaII. The yellow highlight indicates the first exon including the start codon (ATG, in red). The four endonuclease-targeted methylation-sensitive endonuclease recognition sites are highlighted by four different colors. The selected DNA sequence included 2 AciI-sensitive sites (upstream of the start codon), 6 BstUI-sensitive sites (2 upstream and 4 downstream of the start codon), 8 HinP1I-sensitive sites (2 upstream and 6 downstream of the start codon), and 3 HpaII-sensitive sites (2 upstream and 1 downstream of the start codon). The primer regions used for quantitative PCR amplification are underlined; primers were used as follows. Promoter - 5′ UTR: forward = 5′-aga gaa gga tgc gtt gga aggt-3′ and reverse = 5′-gac tcc ggg caa gtg agt gct-3′; exon-intron: forward = 5′-agt cat gcc ctc cta cac ggt ca-3′ and reverse = 5′-agt cat gcc ctc cta cac ggt ca-3′. For the input control, we used the following primers: forward = 5′-tga tgt ggc tgg cct ctt atg tga-3′, reverse = 5′-act ggg act gag tgc agg aaa tgt-3′.

**Figure 2:**
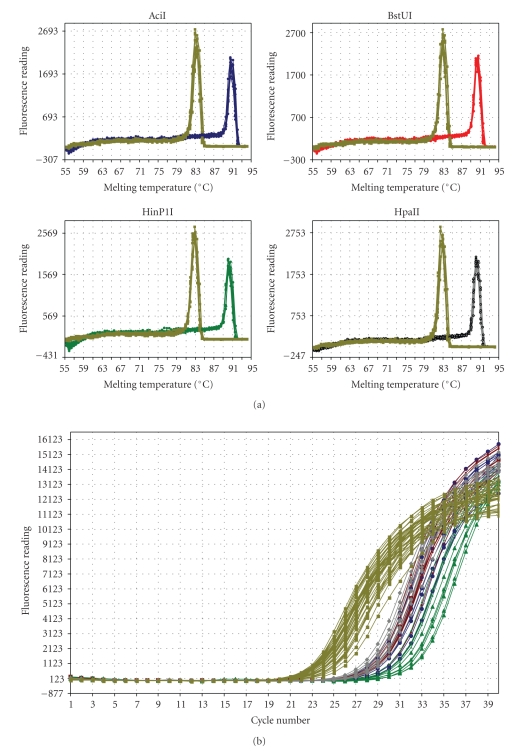
An example of the SYBR Green RD-qPCR assay of 5-LOX DNA methylation (shown are graphs obtained from 10 cerebellar samples). The samples were digested with the methylation-sensitive endonucleases (AciI, BstUI, HinP1I, and HpaII) as described in Material and Methods. The PCR reaction for the promoter-5′UTR (10 samples each: AciI = blue, BstUI = red, HinP1I = green, and HpaII = gray) and corresponding input control regions (shown in yellow) were carried out in separate tubes. Panel A shows the dissociation curve data, which indicate the presence of only one PCR product (peak) for each specific set of primers (fluorescence (first derivative of the raw fluorescence reading multiplied by −1) on the Y-axis versus the PCR product melting temperature (°C) on the X-axis). Panel B shows examples of the amplification plots used for calculating the quantitative data (the amplification plots fluorescence (baseline-corrected raw fluorescence) on the Y-axis versus cycle number on the X-axis). In this assay, the threshold cycle is inversely proportional to the log of the initial copy number. In other words, the more template that is present initially, the fewer the number of cycles required for the fluorescence signal to be detectable above background.

Genomic DNA was extracted from the above-noted tissues. They were homogenized in homogenizing buffer (100 mM NaCl, 10 mM Tris-HCl pH 8.0, 25 mM EDTA, 0.5% SDS, 0.1 mg/ml proteinase K), 100 *μ*l per 10 mg of tissue, and incubated 4-5 hours at 37°C. The DNA was purified with phenol, phenol-chloroform (pH 8.0), precipitated with isopropanol, and dissolved in H_2_O. The DNA methylation assay was designed as follows: The promoter-exon-intron structure of the *Mus musculus* 5-LOX gene was reconstructed by aligning the 5-LOX (ALOX5) mRNA (NM_009662), the promoter, the 5′ untranslated region (UTR), and partial coding region sequences (AF393814) with the mouse genome sequence. This procedure identified a sequence of about 53 kb. Although the mouse 5-LOX gene promoter does not contain typical CpG islands, the mouse 5-LOX promoter-5′UTR-first exon, the adjacent intron portion, and the 3′ end of the gene contain the highest C and G densities. The 5′UTR-first exon and the adjacent intron region (comprising 519 nucleotides) contain 66% of all C and G nucleotides. Furthermore, this region contains 41 CpG dinucleotides; 14 are located upstream from the ATG translation start codon and 27 are downstream (Figure 1). Multiple methylation-sensitive endonuclease recognition sites were located in that portion. Thus, we investigated their methylation rate (i.e., upstream and downstream from the ATG translation start codon) with the aid of four methylation-sensitive endonucleases—AciI (C ↓ CGC), BstUI (CG ↓ CG), HinP1I (G ↓ CGC), and HpaII (C ↓ CGG) (their respective recognition sequences cutting sites are shown in parentheses). To measure the 5-LOX DNA methylation levels, we used the restriction digest-quantitative PCR (SYBR Green RD-qPCR). The selected approach utilizes the ability of methylation-sensitive endonucleases to digest only unmethylated recognition sites, and their inability to act on sites with methylated cytosine. Thus, if the targeted *CpG is methylated, the site is blocked for the enzyme's endonuclease activity and as a result, greater amounts of templates are available for the action of the Taq DNA polymerase. The assay was performed as follows: 1 *μ*g of genomic DNA was used in a restriction digest reaction for each of the four endonucleases. Digested DNA samples were diluted with water and an aliquot (100 ng DNA) was used for qPCR (Stratagene) with the Maxima SYBR Green qPCR Master Mix (Fermentas) according to the manufacturer's protocol. The following primers were used: forward 5′-agagaaggatgcgttggaaggt-3′ and reverse 5′-gactccgggcaagtgagtgct -3′. These primers amplify the 238 nt region upstream of the first ATG translation start codon. This region contains two recognition sites for each of 4 endonucleases selected for the assay. Primers for the exon-intron part were as follows: forward 5′-agtcatgccctcctacacggtca-3′, reverse 5′-agtcatgccctcctacacggtca-3′. They amplify a 344 bp fragment. For the input control, we used the 394 nt region in the first intron because this region does not contain recognition sites for the selected methylation-sensitive endonucleases. This region was amplified with the following primers: forward 5′-tgatgtggctggcctcttatgtga-3′, reverse 5′-actgggactgagtgcaggaaatgt-3′. The qPCR reactions were run with 2 different primer sets (target and input) in separate tubes and the coefficient of variation for the relative amount of a target sequence was calculated [36]. An example of the qPCR assay performance is shown in Figure 2.

### 2.4. Statistics

For statistical analysis, we used SPSS software (version 12.0). Data were analyzed by either one-way ANOVA followed by Dunnett's multiple comparison test, or by an independent sample *t*-test. Results are expressed as the mean ± S.E.M. from 5 to 10 animals. The *P* < .05 values were accepted as statistically significant.

## 3. Results

**Figure 3:**
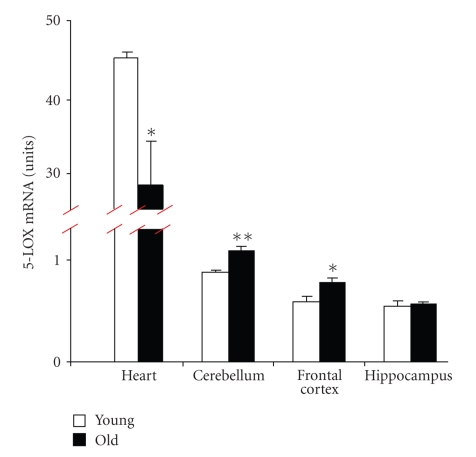
5-LOX mRNA levels in heart and brain tissues of young and old mice. The content of 5-LOX mRNA in the tissues of young (2-month-old, open bars) and old (22-month-old, closed bars) mice was measured by real-time PCR. The results were normalized against the corresponding cyclophilin mRNA contents and are presented as units (the coefficient of variation values amplified by a factor of 1000, see text for details). Data are expressed as mean ± S.E.M (*n* = 5 −10); *P < .05 and **P < .01 versus the corresponding values in young (*t*-test).

Figure 3 shows that the expression levels of 5-LOX mRNA in heart tissue are about fifty times greater than in brain tissue. Similar to previously published data, we found that during aging, 5-LOX content in the brain (e.g., cerebellum and frontal cortex) increases. However, in the heart, aging resulted in significantly decreased 5-LOX mRNA levels (Figure 3). 

**Figure 4:**
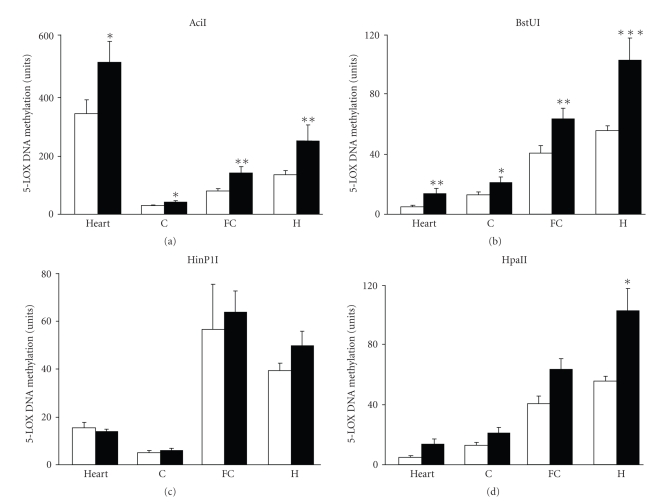
The effect of aging on 5-LOX DNA methylation in the region promoter-5′UTR. The methylation-sensitive endonuclease assay with the four endonucleases—AciI, BstUI, HinP1I, and HpaII—was performed as described in the text and indicated in Figure 1. The heart and brain (C: cerebellum, FC: frontal cortex, H: hippocampus) tissue samples were obtained from 2-month-old mice (open bars) and 22-month-old mice (closed bars). The results are expressed as units (the coefficient of variation values multiplied by a factor of 1000, see text for details) and shown as mean ± S.E.M (heart, C, and H: *n* = 5; FC: *n* = 10). *P < .05, **P < .01, and ***P < .001 versus the corresponding values in samples from 2-month-old mice (*t*-test).

 Using the 5-LOX DNA methylation assay, which is based on the ability of methylation-sensitive endonucleases to digest only unmethylated recognition sites, we found that the four endonucleases—AciI, BstUI, HinP1I, and HpaII—revealed a differential tissue- and brain-region-specific methylation status of 5-LOX DNA (Figure 4). The DNA region targeted by AciI was more methylated in the heart compared to the brain; whereas the DNA regions targeted by BstUI, HinP1I, and HpaII were more methylated in the brain (frontal cortex and hippocampus but not the cerebellum) than in the heart (Figure 4). Aging generally increased 5-LOX DNA methylation but this effect was differential with respect to the DNA region studied, the type of the tissue/organ (e.g., heart versus brain), and the brain region we investigated. Focusing on the promoter-5′UTR region (Figure 4), we found the effect of aging in the areas targeted by AciI, BstUI, and HpaII but not HinP1l. Thus, the methylation levels assayed by AciI and BstUI were increased by aging in all tissues studied, whereas the methylation levels studied by HpaII were increased only in the hippocampus. Focusing on the exon-intron region, we found that aging increased methylation only in the brain and only in the DNA region sensitive to BstUI (units as in Figure 4 (two-tail *t*-test); frontal cortex: young = 110 ± 14, old = 163 ± 14, *P* < .05, *n* = 10; hippocampus: young = 86 ± 8, old = 136 ± 17, *P* < .05, *n* = 5; cerebellum: young = 36 ± 6, old = 58 ± 10, n.s., *n* = 5; heart: young = 42 ± 20, old = 54 ± 10; ns, *n* = 5). 

**Figure 5:**
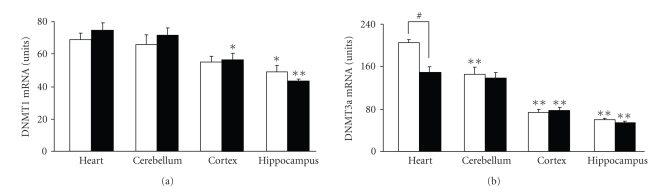
DNMT1 (a) and DNMT3a (b) mRNA levels in heart and brain tissues of young and old mice. The content of DNMT1/DNMT3a mRNA in the tissues of young and old mice was measured by real-time PCR. The results were normalized against the corresponding cyclophilin mRNA contents and are presented as units (the coefficient of variation values amplified by a factor of 1000, see text for details). Data are expressed as mean ± S.E.M (*n* = 5 −10). Note that the only aging-associated difference was observed for DNMT3a mRNA content in the heart (^#^ *P* < .01, *t*-test). The content of DNMT mRNA was generally greater in the heart compared to the brain; *P < .05 and *P < .001 versus the corresponding values in the heart in the same age group (Dunnett's test).

 The content of mRNA for DNMT1 and DNMT3a (*maintenance* and the *de novo* DNMTs, resp.) was generally greater in the heart compared to the brain, particularly the content of DNMT3a mRNA (Figure 5). The only aging-associated difference we observed was the decreased DNMT3a mRNA content in the heart of old versus young mice (Figure 5(b)).

## 4. Discussion

In line with previous reports [14], we confirmed that 5-LOX mRNA is expressed in the mouse brain, albeit at levels significantly lower than 5-LOX mRNA levels measured in heart tissue. We also confirmed that aging increases 5-LOX mRNA content in the brain. In our previous work with rat brain tissue, we found an aging-associated 5-LOX increase in the cerebellum and the hippocampus [37]. In this study with mice, we found an aging-associated 5-LOX increase in the cerebellum and the frontal cortex but not in the hippocampus. These differences do not appear to be species-related (e.g., rat versus mouse) because others found that aging increases 5-LOX expression in the mouse hippocampus but not in the cerebellum and cortex [14]. The reason for these discrepancies is not apparent, but considering the putative epigenetic regulation of 5-LOX expression, it is likely that individual life experiences of various colonies of old rodents could result in a colony-specific aging-modified epigenetic regulation of 5-LOX expression. Regardless of the observed regional differences, all previous studies [14, 17, 37] and our present results are consistent as to the direction (i.e., increase) of aging-associated changes in brain 5-LOX expression. In contrast to the observed effects of aging in brain tissue, we found that in heart tissue aging was associated with a decrease in 5-LOX mRNA content. To our knowledge, this is the first time that heart 5-LOX expression has been investigated in the context of aging. Previous studies reported that 5-LOX content in the heart remains stable during cardiac remodeling [16] but can be altered by dietary modifications [22]. On the other hand, 5-LOX protein content in the aorta was significantly increased in old (24 months) compared to adult (6 months) rats [38]. 

 DNA methylation is an epigenetic mechanism involved in the regulation of gene expression including the effects of aging and tissue-specific expression. We observed that in the heart, which expresses about 50 times greater 5-LOX mRNA levels than the brain, the DNA regions targeted by BstUI, HinP1I, and HpaII were less methylated than those in the brain. Typically, methylation of proximal promoters containing a high density of CpG dinucleotides (CpG islands) leads to long-term gene silencing. 

 Compared to human 5-LOX, the mouse 5-LOX DNA sequence lacks the typical CpG islands. The methylation status of the nonisland CpG loci appears to be equally relevant for gene regulation [39]. For example, Sakamoto et al. [40] reported that the tissue-dependently and differentially methylated regions were disproportionately distributed in the nonisland CpG loci and that these loci were located not only in 5′ regions of genes but also in intronic and nongenic regions. The mouse 5-LOX promoter-5′ UTR sequence has an equal number (i.e., 2) of recognition sites for each of the four endonucleases used in our assay. Nevertheless, the effect of aging on this 5-LOX DNA sequence was extremely heterogeneous. For example, aging did not affect the methylation status assayed at the HinP1I sites, it only affected the HpaII sites in the hippocampus and produced a similar effect on AciI and BstUI sites in all tissue samples studied. On the other hand, the exon-intron sequence has 4 BstUI, 6 HinP1I, 1 HpaII, and 1 AciI site. However, the only effect of aging in this 5-LOX DNA sequence was observed in brain tissues and only in BstUI sites. 

 The pattern of DNA methylation depends on enzymes such as DNMTs, in particular DNMT1 which is involved in the maintenance of DNA methylation patterns and DNMT3a which predominantly contributes to de-novo DNA methylation. Senescence has been associated with alterations in DNMT activity [41] and expression [28]. Although the expression of DNMT1 and DNMT3a showed some tissue and regional differences, we did not observe major aging-associated alterations in the mRNA levels of these two enzymes, with the only exception that there was lower DNMT3a mRNA content in the heart of old mice. However, this finding does not exclude the possibility of aging-altered DNMT activity in the brain. Furthermore, multiple enzymatic mechanisms are involved in determining the pattern of DNA methylation, including DNA-demethylases [42]. For example, recent findings indicate that neuronal activity is capable of triggering epigenetic DNA demethylation [43], and it remains to be investigated whether this process is affected by aging and whether it could alter 5-LOX DNA methylation in the absence of significant DNMT changes. 

 Further research is needed to elucidate if the observed changes in 5-LOX DNA methylation could play a role in aging-associated modifications of tissue 5-LOX mRNA levels. For such a functional role, DNA methylation status would have to influence the binding of various methylation-sensitive regulatory factors (e.g., transcription activators and repressors). We subjected the 5-LOX DNA sequences used in our assay (Figure 1) to an analysis with the software for searching transcription factor binding sites (TFBIND), which uses the weight matrix in transcription factor database TRANSFAC R.3.4. [44]. This analysis, along with the survey of available literature, suggests that in the region upstream of the mouse 5-LOX ATG start codon, the likely candidates are the following transcription factors: AHR/ARNT, NMYC, E2F, AP4, AP2, USF, and SP1. In the coding region, putative regulatory factors include AHR/ARNT, USF, ELK1, HSF1, HSF2, ARP1, RFX1, GATA1, AP4, E2F, and ARP1. Nevertheless, one must take into account that the tissue samples we used in this study contained a mixture of cell types and that the link between 5-LOX mRNA and 5-LOX DNA methylation may depend on the cell type. Inherent in studies with tissue samples as opposed to research in cell cultures is that 5-LOX DNA extracted from tissue samples derives from both 5-LOX mRNA-expressing and mRNA-nonexpressing cells. This technical limitation negatively impacts the ability to directly relate mRNA content and DNA methylation status. Similarly, cell type may be decisive in determining the type of 5-LOX metabolites produced (e.g., leukotrienes versus lipoxins). 

 Although leukotriene synthesis in the CNS and in the heart can occur via a transcellular biosynthetic pathway that is believed not to require 5-LOX expression [12, 13], aging-increased leukotriene synthesis in the hippocampus [14] and aorta [38] appears to be associated with increased 5-LOX expression. It was proposed that in the CNS, metabolites derived from 5-LOX activity could influence neural plasticity, such as the expression of hippocampal long-term depression [10]. Furthermore, it has been established that the biological effects of leukotrienes in the CNS [8] and heart [23] can be counteracted by noninflammatory 5-LOX metabolites lipoxins. No data are available on the effects of aging on CNS and heart production of lipoxins. This information appears necessary for a full understanding of the biological implications of aging-altered expression of 5-LOX in the brain and heart. 

 In conclusion, we found that in addition to altering 5-LOX mRNA content in heart and brain tissues, aging increased 5-LOX DNA methylation, and also that this effect was DNA-site- and tissue-specific. It has to be stressed that our results were obtained in tissue samples containing a mixture of various cell types and that further insight into functional implications of DNA methylation for 5-LOX mRNA expression would require cell-type directed studies. For example, one possibility is to focus on neuronal DNA in brain tissue samples by applying methods for neuronal nuclei isolation [45].

## Figures and Tables

**Table 1:** Primer sequences used to assay 5-LOX, DNMT1, DNMT3a, and cyclophilin (reference) mRNA.

mRNA	Gene bank accessio number	Primers	Fragment size (bp)
5-LOX	NM_009662	F: 5′-ATTGCCATCCAGCTCAACCAAACC-3′	178
		R: 5′-TGGCGATACCAAACACCTCAGACA-3′	
DNMT1	NM_010066	F: 5′-AGTGCAAGGCGTGCAAAGATATGG-3′	150
		R: 5′-TGGGTGATGGCATCTCTGACACAT-3′	
DNMT3a	NM_007872	F: 5′-ACAAGAATGCTACCAAAGCAGCCG -3′	200
	NM_153743	R: 5′-TGAGAACTTGCCATCTCCGAACCA-3′	
Cyclophilin	X52803	F: 5′- AGCATACAGGTCCTGGCATCTTGT –3′	153
		R: 5′- AAACGCTCCATGGCTTCCACAATG -3′	
